# All-*Trans*-Retinoic Acid Suppresses Neointimal Hyperplasia and Inhibits Vascular Smooth Muscle Cell Proliferation and Migration *via* Activation of AMPK Signaling Pathway

**DOI:** 10.3389/fphar.2019.00485

**Published:** 2019-05-09

**Authors:** Jingzhi Zhang, Bo Deng, Xiaoli Jiang, Min Cai, Ningning Liu, Shuangwei Zhang, Yongzhen Tan, Guiqiong Huang, Wen Jin, Bin Liu, Shiming Liu

**Affiliations:** ^1^Guangzhou Institute of Cardiovascular Disease, Guangdong Key Laboratory of Vascular Diseases, State Key Laboratory of Respiratory Disease, The Second Affiliated Hospital of Guangzhou Medical University, Guangzhou, China; ^2^Department of Traditional Chinese Medicine, The Second Affiliated Hospital of Guangzhou Medical University, Guangzhou, China; ^3^Department of Internal Medicine, Huizhou Hospital of Traditional Chinese Medicine, Huizhou, China; ^4^Department of Cardiology, Guangdong Second Provincial General Hospital, Guangzhou, China

**Keywords:** all-*trans*-retinoic acid, neointimal hyperplasia, vascular smooth muscle cell, proliferation, AMP-activated protein kinase

## Abstract

The proliferation and migration of vascular smooth muscle cells (VSMC) is extensively involved in pathogenesis of neointimal hyperplasia. All-*trans*-retinoic acid (ATRA) is a natural metabolite of vitamin A. Here, we investigated the involvement of AMP-activated protein kinase (AMPK) in the anti-neointimal hyperplasia effects of ATRA. We found that treatment with ATRA significantly reduced neointimal hyperplasia in the left common carotid artery ligation mouse model. ATRA reduced the proliferation and migration of VSMC, A7r5 and HASMC cell lines. Our results also demonstrated that ATRA altered the expression of proliferation-related proteins, including CyclinD1, CyclinD3, CyclinA2, CDK2, CDK4, and CDK6 in VSMC. ATRA dose-dependently enhanced the phosphorylation level of AMPKα (Thr172) in the left common carotid artery of experimental mice. Also, the phosphorylation level of AMPKα in A7r5 and HASMC was significantly increased. In addition, ATRA dose-dependently reduced the phosphorylation levels of mTOR and mTOR target proteins p70 S6 kinase (p70S6K) and 4E-binding protein 1 (4EBP1) in A7r5 and HASMC. Notably, the inhibition of AMPKα by AMPK inhibitor (compound C) negated the protective effect of ATRA on VSMC proliferation in A7r5. Also, knockdown of AMPKα by siRNA partly abolished the anti-proliferative and anti-migratory effects of ATRA in HASMC. Molecular docking analysis showed that ATRA could dock to the agonist binding site of AMPK, and the binding energy between AMPK and ATRA was -7.91 kcal/mol. Molecular dynamics simulations showed that the binding of AMPK-ATRA was stable. These data demonstrated that ATRA might inhibit neointimal hyperplasia and suppress VSMC proliferation and migration by direct activation of AMPK and inhibition of mTOR signaling.

## Introduction

Atherosclerosis is a chronic inflammatory disorder occurring in the arterial walls of large and medium-sized arteries, accounting for one of the most common causes of morbidity as well as mortality globally (Go et al., 2014). Percutaneous coronary intervention (PCI), also known as coronary angioplasty, is commonly used to treat myocardial infarction, although the application of PCI remains limited because of restenosis after angioplasty (Pleva et al., 2018). Neointimal hyperplasia is an important pathological characteristic of restenosis after angioplasty (Zhang et al., 2008). It is well-established that the proliferation and migration of VSMC plays a central role of neointimal hyperplasia (Curcio et al., 2011). Therefore, inhibition of VSMC proliferation is an effective strategy against restenosis after angioplasty.

All-*trans*-retinoic acid, a natural derivative of vitamin A, exerts therapeutic functions for cardiovascular disease. For instance, it has been reported that treatment with ATRA significantly inhibits the formation of atherosclerotic lesions in the high fat diet-induced atherosclerosis rabbit model (Zhou et al., 2012). It has also been found that ATRA inhibits restenosis after balloon angioplasty in the atherosclerotic rabbit (Wiegman et al., 2000). Moreover, a previous study has demonstrated that treatment with ATRA inhibits the proliferation VSMC by up-regulating the expression of Klf4 (Wang et al., 2008).

AMP-activated protein kinase, a physiological sensor of cellular energy status, widely participates in carbohydrate metabolism, lipid metabolism, aging, cell growth and protein metabolism (Steinberg and Schertzer, 2014; Day et al., 2017). AMPK exists as a heterotrimeric complex that comprises a catalytic subunit, AMPKα, and two regulatory subunits, AMPKβ and γ (Viollet et al., 2010). The AMPKα subunits contain conventional kinase domains at the N terminus and could be phosphorylated at Thr172. It has recently become clear that AMPK plays a crucial role in atherosclerosis, especially in regulating neointimal hyperplasia and VSMC proliferation (Ferri, 2012). Deletion of AMPKα promoted neointimal hyperplasia by enhancing VSMC proliferation and migration (Song et al., 2011). Additionally, the significance of AMPK in vascular function is well-supported by anti-atherosclerosis agents, including statins, thiazolidinediones, leptin and rosiglitazone, which exhibit anti-atherosclerosis effects, at least partially, through the activation of AMPK (Zou and Wu, 2008). In addition, activation of AMPK further results in inhibition of mTOR signaling in a variety of cells, including VSMC (Ke et al., 2016). Therefore, targeting AMPK might be a good strategy for atherosclerosis management. A previous study has found that ATRA enhances the activity of AMPK in ovarian cancer, skeletal muscle cells and endothelial cells (Ke et al., 2016). Nevertheless, it remains unclear whether ATRA inhibit ligation-induced neointimal hyperplasia and VSMC proliferation *via* activation of AMPK. Based on the previous reports, it is worthwhile to investigate the effect of ATRA on increasing AMPK activity in VSMC. To fill this gap in knowledge, we used A7r5 and HASMC VSMCs as the *in vitro* model and common carotid artery ligation as the *in vivo* model to investigate whether AMPK is involved in the anti-proliferative and anti-migratory effects of ATRA. Molecular docking and molecular dynamics (MD) simulations were used to examine the interactions between AMPK and ATRA.

## Materials and Methods

### Chemicals and Antibodies

ATRA was commercially purchased from Sigma Chemical (St. Louis, MO, United States). Compound C was obtained from Selleckchem (Shanghai, China). GAPDH antibody was purchased from Abcam (Cambridge, United Kingdom). Phospho-AMPKα, AMPKα, phospho-mTOR, mTOR, phospho-p70S6K, phospho-4EBP1 CyclinD1, CyclinD3, CyclinA2, CDK2, CDK6, CDK4, Bax, Bcl-xl, cleaved-caspase3 and Pro-caspase3 antibodies were commercially purchased from Cell Signaling Technology (Beverly, MA, United States). LIVE/DEAD Assay Kit was obtained from Invitrogen. Besides, other reagents were also purchased from commercial sources.

### Animals

Male C57BL/6 mice (aged 8–10 weeks) were obtained from the Guangdong Laboratory Animal Monitoring Institute (Specific pathogen-Free, Certificate No. SCXK-2013-0002). We performed all the procedures involving laboratory animals in accordance with the guidelines of the Institutional Animal Care and Use Committee of The Second Affiliated Hospital of Guangzhou Medical University. Before conducting the experiment, mice were allowed to acclimatize to the new environment for 1 week.

### Common Carotid Artery Ligation

In brief, animals were anesthetized by exposure of 3% isoflurane in 100% oxygen. The left common carotid artery was dissected from the surrounding tissue under a microscope, and ligated near its bifurcation by using 6–0 silk ligature. In sham groups, the left common carotid artery was dissected from the surrounding tissue without subsequent ligation. Three days later, mice were randomly assigned into four groups (*n* = 8) and were treated as follows: Sham group (administration with hydration medium: 30% PEG400, 5% Tween 80, and 5% DMSO), Ligation group (common carotid artery ligation and administration with hydration medium), and ATRA groups (common carotid artery ligation and administration with ATRA 10 mg/kg and 20 mg/kg). Twenty-one days after surgery, animals were anesthetized and perfused with PBS first, followed by 4% paraformaldehyde. Carotid arteries were excised, and then embedded in paraffin. Cross-sections (5 μM) were taken starting at the ligation site and stained with hematoxylin and eosin and immunohistochemical staining.

### Cell Culture

A7r5 was purchased from Cell Bank of Type Culture Collection of Chinese Academy of Sciences (Shanghai, China). HASMC was purchased from ScienCell Research Laboratories (Carlsbad, CA, United States). These cell lines were cultured in Dulbecco’s modified Eagle’s medium (4.5 g/L glucose, DMEM) containing 10%fetal bovine serum (FBS) as well as 100 μg/mL penicillin/streptomycin at 37°C in a humidified atmosphere containing 5% CO_2_. Cells were seeded in 60 mm dishes at an initial density of 1 × 10^5^ cells/well and grown to approximately 80% confluence. For the siRNA transfection experiments, VSMC were grown to 60% confluence and AMPK α1/2 siRNA mix or negative control (NC) siRNA (Santa Cruz, CA, United States) were transfected using Lipofectamine 3000 (Invitrogen, CA, United States) according to the manufacturer’s instructions.

### MTS Cell Proliferation Assay

MTS assay kit (Promega Corporation, Madison, WI, United States) was used to test cell proliferation as previously described (Liu et al., 2016). Briefly, after different stimulations, culture medium was aspirated from 96-well plates. The MTS reagent was added into 0.1% FBS in a ratio of 1:5, and uniformly mixed, followed by addition of 100 μL mixture into each well. After additional incubation at 37°C in a humidified atmosphere with 5% CO_2_ for 2 h, the absorbance at 490 nm was detected by a 96-well plate reader. The calculation of cell proliferation rate was in line with the manufacturer’s instruction using the following formula: cell proliferation rate = [OD (Experiment) - OD (blank)]/[OD (Control) - OD (blank)] × 100%.

### EdU Proliferation Assay

The cells were incubated with 5-ethynyl-2-deoxyuridine (EdU) (100 μM, Cell Light EdU DNA imaging Kit, Guangzhou RiboBio, China) for an additional 6 h. Subsequently, cell staining was conducted according to standard protocol. Briefly, after discarding the EdU medium mixture, 4% paraformaldehyde was added to fix cells for 30 min at room temperature, followed by washing with glycine (2 mg/mL) for 5 min. After addition of 0.2% Trion X-100 for 10 min, the cells were washed with PBS twice, followed by addition of click reaction buffer for 10–30 min in the dark. Then, the cells were washed with 0.5% Triton X-100 for three times, and stained with Hoechst33342 (5 μg/ml) at room temperature for 30 min. After washing with 0.5% Triton X-100 for five times, 150 μL PBS was added to the wells. EdU staining images were photographed under fluorescent microscope (Nikon, Ti-s), and the proportion of EdU positive cells was obtained using the formula: (EdU add-in cells/Hoechst stained cells) × 100%.

### Live/Dead Cell Staining

Cell death was detected by the LIVE/DEAD Assay Kit (Invitrogen). Live and dead cells were distinguished by using AM (live cells, labeled with green) or ethidium homodimer-1 (dead cells, labeled with red) probes. Fluorescence imaging of the cells was taken with fluorescence microscopy (Nikon Eclipse Ti-S).

### Transwell Migration Assay

Transwell migration assay was used to measure the migratory ability of VSMCs. Briefly, A7r5 and HASMC VMSCs were seeded into the upper Transwell chamber (1 × 10^5^ cells/well) and treated with different condition with serum-free DMEM. The lower Transwell chambers were filled with DMEM with 10% FBS and placed in 24-well plates. After 24 h, the non-migrating cells in the upper Transwell chamber were removed with cotton swab. Then the Transwell chambers were washed with PBS for three times; the lower chamber was fixed with 4% paraformaldehyde. Next, the migrated cells were stained with crystal violet for 15 min. Five visual fields were randomly selected from each Transwell chamber and captured at 100× magnification under an microscopy (Leica, S40). The number of migrated cells were counted using the ImageJ software.

### Western Blot Assay

Western blot analysis was performed as previously described (Liu et al., 2017). Briefly, after washing with ice-cold PBS, RIPA buffer (Beyotime Institute of Biotechnology) with phosphatase as well as protease inhibitors (cocktail tablet; Roche Applied Science) was utilized to lyse A7r5 and HAMSC cells. After centrifuging at 12,000 ×*g* at 4°C for 15 min, supernatants were collected, followed by the determination of protein concentration of each sample using BCA protein assay kit (Thermo) according to the manufacturer’s instruction. Protein samples (10 μg) were subjected to SDS-PAGE, and subsequently transferred onto PVDF membrane (Millipore, Bedford, MA, United States). After blocking in 5% non-fat milk, the membranes were incubated with indicated primary antibodies overnight (p-AMPKα, p-mTOR, AMPKα, mTOR, p-p70SK6, p-4EBP1, CyclinD1, CyclinD3, CyclinA2, CDK2, CDK4, CDK6, Bax, Bcl-xl, cleaved-caspase3 and Pro-caspase3, all at a dilution of 1:1000). And GAPDH was used as the internal control (dilution 1:5000). Finally, ImageJ software was used to quantify the band intensity, followed by normalization with the intensity of internal control.

### Molecular Simulation Study

Molecular docking was performed to clarify the binding mechanism between AMPK (PDB ID:4QFR) and ATRA (ZINC ID:12358651). The crystal structure of AMPK was co-crystallized with A-769662, an AMPK agonist (Calabrese et al., 2014). The binding domain of A-769662 in AMPK is between AMPK α and β heterodimer (Calabrese et al., 2014). Therefore, we removed AMPKγ and investigated the interaction between ATRA and AMPK α-β heterodimer in A-769662 binding domain. The protein for molecular docking simulation was prepared by removing water molecules and bound ligands. Energy minimization of ligands was performed by YASARA. Autodock Vina (Scripps Research Institute, United States) was utilized for molecular docking (Trott and Olson, 2010). The best conformations were taken as the starting conformation for MD simulation.

Molecular dynamics simulation was performed with YASARA (Land and Humble, 2018). AMBER 03 forcefield was used to run all simulations. Specifically, 0.9% NaCl served as solvation of the receptor-ligand complex in a dodecahedron box, with a distance of 5 Å between the box and the solute. The initiation of simulated annealing minimizations was set at 298 K, with velocities scaling down by 0.9 every 10 steps lasting for 5 ps. Following energy minimization, temperature of the system was adjusted utilizing using Berendsen thermostat to minimize the influence of temperature control. In addition, velocities were rescaled only every 100 simulation steps, whenever the mean of the last 100 detected temperatures converged. Finally, 100 ns MD simulations were conducted at a rate of 2 fs, and the coordinates of the complexes were saved every 10 ps.

### Statistical Analysis

The SPSS software (version 13.0, SPSS Inc., Chicago, IL, United States) was used for data analysis; data are expressed as mean ± SD. One-way ANOVA was utilized for univariate analysis. The least significance difference (LSD) test was used for analyzing the differences between two groups. The comparison of mean values was carried out using Welch’s test in case of non-homogenous variances. In addition, Dunnett’s T3 was used to determine the differences between two groups. *P* < 0.05 was considered as statistically significant.

## Results

### ATRA Inhibited Neointimal Hyperplasia and Suppressed the Proliferation and Migration of VSMCs

**FIGURE 1:**
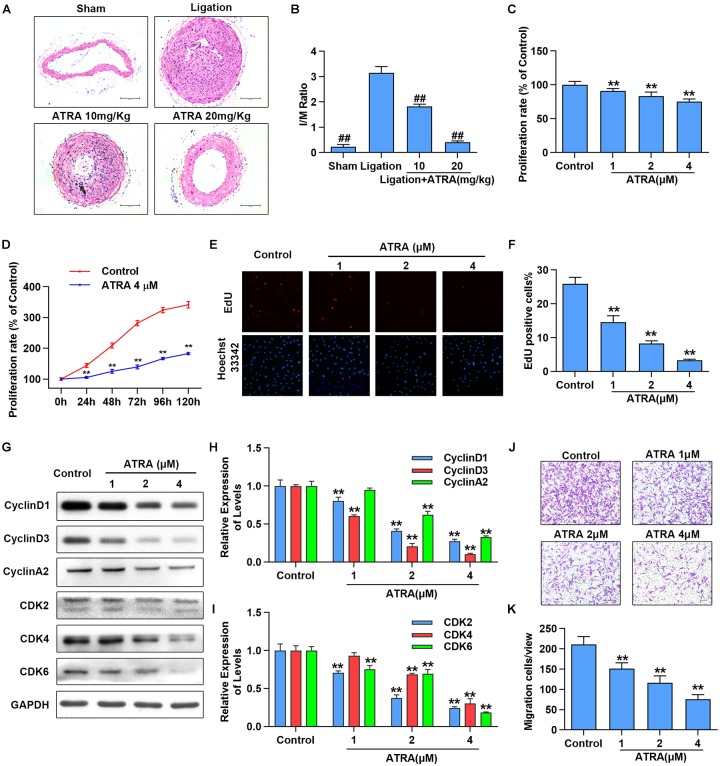
ATRA inhibited neointimal hyperplasia and suppressed the proliferation and migration of VSMC. **(A)** Representative sections of the left common carotid arteries from the sham group, ligation group and ATRA-treated groups by hematoxylin–eosin staining. **(B)** Morphometric quantification of intimal/media (I/M) ratio (*n* = 8). **(C)** A7r5 cells were incubated with the indicated doses of ATRA (1, 2 as well as 4 μM) for 24 h, followed by the MTS assay to determine the proliferation of A7r5 (*n* = 6). **(D)** A7r5 cells were treated with ATRA (4 μM) for 0, 24, 48, 72, 96, and 120 h, and MTS assay was performed to test cell proliferation rate (*n* = 6). **(E)** Representative images of EdU staining. EdU (in red) stained the regions of cell proliferation; Hoechst33342 (in blue) stained the nuclei. **(F)** Percentage of EdU positive cells of A7r5 (*n* = 3). **(G)** The expressions of CyclinD1, CyclinD3, CyclinA2, CDK2, CDK4, and CDK6 were tested via western blotting. **(H)** Relative levels of CyclinD1, CyclinD3, and CyclinA2 (*n* = 3). **(I)** Relative expression levels of CDK2, CDK4, and CDK6 (*n* = 3). **(J)** A7r5 cells were treated with different concentrations of ATRA (1, 2, and 4 μM), and tested by performing Transwell assays for 12 h. **(K)** The number of cells in each field of view (*n* = 5). Data are presented as mean ± SD. ^#^*p* < 0.05, ^##^*p* < 0.01 compared with the ligation group. ^∗^*p* < 0.05, ^∗∗^*p* < 0.01 compared with the Control group.

To investigate the effects of ATRA on neointimal hyperplasia, we first checked the influences of ATRA treatment *in vivo* on neointimal formation in common carotid artery ligation mice. After being treated with ATRA for 28 days, we observed that the ratio of intimal/media (I/M) was significantly enhanced by common carotid artery ligation, while I/M were dose-dependently decreased by ATRA treatment (Figure 1A,B). To determine if ATRA inhibited the proliferation of VSMC, A7r5 rat VSMC cell line was treated with ATRA for 24 h, followed by MTS assay and EdU staining to detect the proliferation of A7r5. As shown in Figure 1C, treatment with ATRA (1, 2, and 4 μM) inhibited the proliferation rate (% of Control) of VSMC in a dose-dependent pattern. Then, we investigated whether there exists a time-dependent mechanism involving ATRA in the inhibition of VSMC proliferation by treatment with ATRA (4 μM) for 0, 24, 48, 72, 96, and 120 h, and we detected cell proliferation by MTS. As shown in Figure 1D, ATRA time-dependently decreased the proliferation rate (% of 0 h) of A7r5. Moreover, the inhibitory effects of ATRA on VSMC proliferation were also determined by EdU staining (Figure 1E,F). Consistently, the EdU positive cell number was dose-dependently reduced under ATRA treatment. Next, we detected the expression levels of cell cyclin proteins, including CyclinD1, CyclinD3, and CyclinA2, and cyclin-dependent kinases, CDK2, CDK4, and CDK6. As shown in Figure 1G,I, the expression levels of CyclinD1, CyclinD3, CyclinA2, CDK2, CDK4, and CDK6 were significantly reduced following ATRA administration in a dose-dependent way. Next, to further investigate the effect of ATRA on the migration of VSMC, the Transwell assays were performed. As shown in Figure 1J,K, ATRA inhibited A7r5 cell migration in a dose-dependent manner.

A7r5 is a rat VSMC cell line. To increase the translational potential of the study, we repeated some key experiment in a human VSMCs line, namely HASMC. As shown in Supplementary Figures S1A–C, we found that this compound reduced the cell proliferation of HASMC, in a dose-dependent manner, based on MTS and EdU assays. We also demonstrated via western blotting that ATRA inhibited the expression of proliferation-related proteins including CyclinD1, CyclinD3 and CyclinA2 in a dose-dependent manner (Supplementary Figures S1D,E). As shown in Supplementary Figures S1F,G, we found ATRA dose-dependently suppressed the cell migration of HASMC. These results suggested that treatment with ATRA inhibited neointimal hyperplasia and suppressed proliferation and migration of VSMCs. These results indicated that ATRA inhibited VSMC proliferation and migration in a dose-dependent manner.

During neointima formation, various growth factors including PDGF-BB might enhance the proliferation and migration of VSMCs. Thus, we investigated whether ATRA could inhibit PDGF-BB-induced VSMC proliferation and migration. As shown in Supplementary Figures S2A,B, the proliferation rate (% of Control) of A7r5 and HASMC were raised by PDGF-BB, while ATRA reduced the proliferation rate of these two VSMC cell lines in a dose-dependent manner. Moreover, the inhibitory effects of ATRA on VSMC migration were determined by the Transwell assays (Supplementary Figures S2C–E). Consistently, the migratory ratio was increased by PDGF-BB and dose-dependently reduced under ATRA treatment. These results suggested that ATRA inhibited PDGF-BB-induced VSMC proliferation and migration.

To determine whether ATRA induced cell injury in VSMCs, we performed the live-dead cell staining and detected the expression of apoptosis-relative proteins via western blotting. Live-dead cell staining results are presented in Supplementary Figure S3A, live cells are marked with green and dead cells with red. We found that ATRA reduced cell density of live cells while dead cells were not found in ATRA treated groups. Then, we detected the expression levels of apoptosis-related protein, including Bax, Bcl-xl, cleaved-caspase3 and pro-caspase3. As shown in Figure 1H,I, the expression of Bax, Bcl-xl cleaved-caspase3 and pro-caspase3 were not changed by ATRA (Supplementary Figures S3B,C).

### ATRA Enhanced the Activation of AMPK *in vivo* and *in vitro*

**FIGURE 2:**
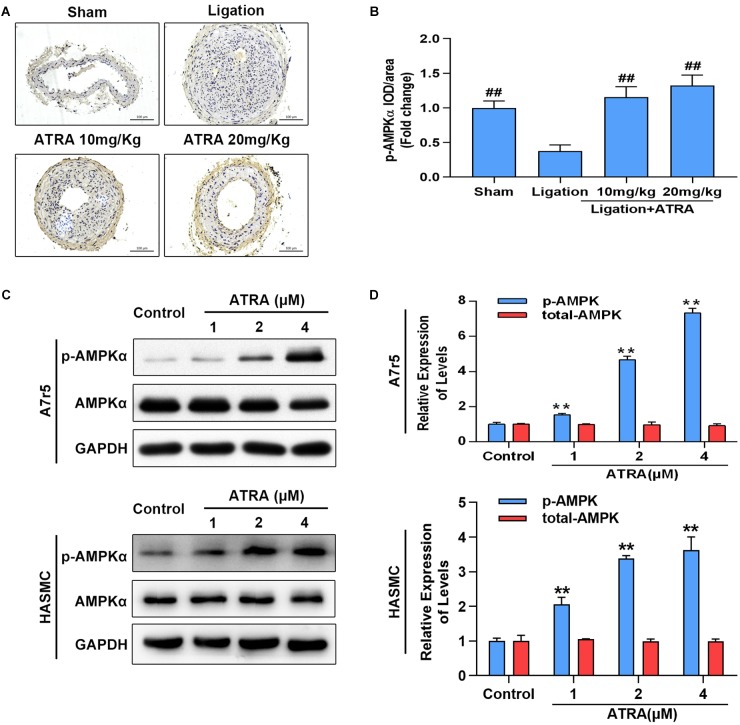
ATRA dose dependently enhanced AMPKα activation. **(A)** Representative image for the p-AMPKα immunohistochemical staining in the left common carotid arteries sections from the sham group, ligation group, and ATRA-treated groups. **(B)** Quantification of immunohistochemical staining-positive areas in the left common carotid arteries sections from the sham group, ligation group, and two ATRA-treated groups (*n* = 8). **(C)** A7r5 and HASMC cells were separately incubated with the indicated doses (1, 2 as well as 4 μM) of ATRA for 6 h, levels of p-AMPKα and AMPKα in total lysates were assessed by western blotting. **(D)** Relative expression levels of p-AMPKα and total AMPKα (*n* = 3). Data are shown as mean ± SD. ^#^*p* < 0.05, ^##^*p* < 0.01 compared with the ligation group. ^∗^*p* < 0.05, ^∗∗^*p* < 0.01 compared with the Control group.

Here, we investigated whether ATRA enhances AMPK activation *in vivo* and *in vitro*. Ligation of common carotid artery significantly reduced the phosphorylation level of AMPKα, while administration of ATRA (10 mg/kg and 20 mg/kg) significantly enhanced the phosphorylation level of AMPKα (Figure 2A,B). Accordingly, in A7r5 culture cell model, ATRA (1, 2, and 4 μM) significantly enhanced the phosphorylation level of AMPKα at Thr172 in a dose-dependent pattern, without altering the total level of AMPKα in A7r5 and HASMC VSMCs (Figure 2C,D). Next, we compared the anti-proliferative and anti-migratory effects between ATRA and AMPK agonist, AICAR. Western blot results are shown in Supplementary Figures S4A,B. For ATRA treatment (4 μM), AICAR (1 mM) significantly enhanced the phosphorylation level of AMPKα in HASMC. MTS and Transwell assays demonstrated that both ATRA and AICAR could inhibit VSMC proliferation and migration (Supplementary Figures S4C–E). Our data indicates that ATRA has similar effects with AICAR.

### ATRA Reduced the Activation of mTOR Signaling Pathway

**FIGURE 3:**
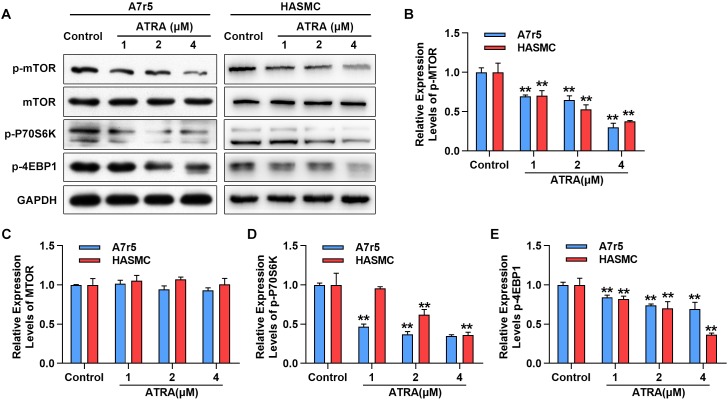
ATRA suppressed mTOR signaling pathway. **(A)** A7r5 and HASMC cells were incubated with the indicated doses (1, 2 as well as 4 μM) of ATRA for 6 h, **(B)** levels of p-mTOR, mTOR, p-p70S6K as well as p-4EBP1 were tested via western blotting. **(C)** Relative expression levels of p-mTOR and total-mTOR. **(D)** Relative expression level of p-p70S6K (*n* = 3). **(E)** Relative expression level of p-4EBP1 (*n* = 3). Data are shown as mean ± SD. ^∗^*p* < 0.05, ^∗∗^*p* < 0.01 compared with the Control group.

Next, we further determined whether ATRA inhibits the activation of mTOR signaling molecules in A7r5 and HASMC, including rapamycin (mTOR), p70 S6 kinase (p70S6K) and 4EBP1, by western blotting. As shown in Figure 3A–C, administration with ATRA inhibited the phosphorylation level of mTOR in a dose-dependent way without changing the expression of total mTOR. Consistently, the phosphorylation levels of p70S6K as well as 4EBP1 were dose-dependently reduced by ATRA (Figure 3A,D,E). Our findings suggested that ATRA inhibited the activation of mTOR signaling pathway.

### Inhibition of AMPK Partly Abolished Anti-proliferative and Anti-migratory Action of ATRA in VSMCs

**FIGURE 4:**
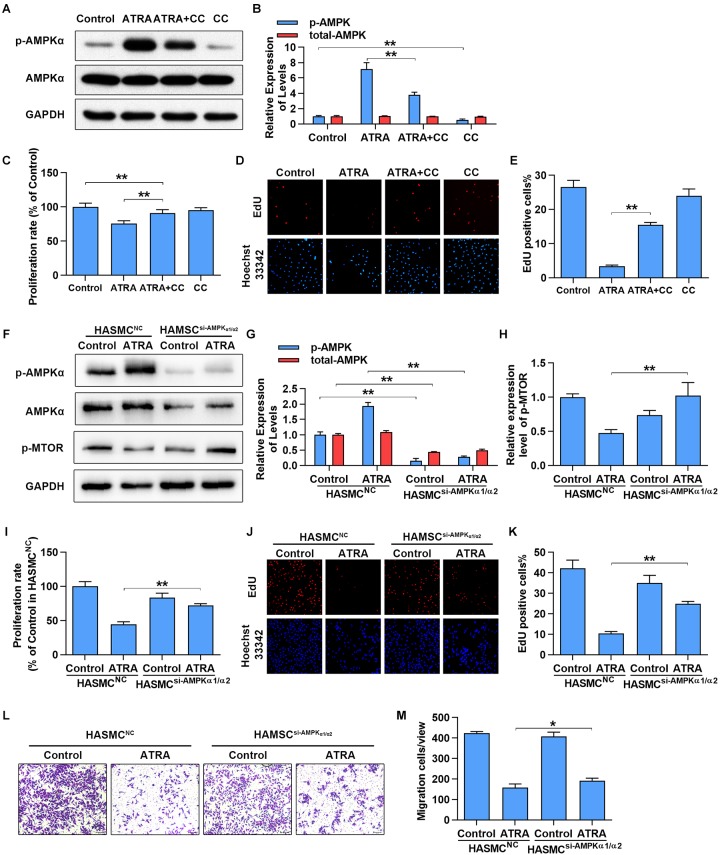
Inhibition of AMPKα partly abrogated the anti-proliferation and anti-migration effects of ATRA. **(A)** A7r5 cells were cultured with ATRA (4 μM) alone or co-treated with ATRA (4 μM) as well as AMPKα inhibitor (CC, 5 μM) for 6 h, then the expression levels of p-AMPKα and AMPKα were assessed via western blotting. **(B)** Relative expression levels of p-AMPKα and total-AMPKα (*n* = 3). **(C)** A7r5 cells were cultured with ATRA (4 μM) alone or co-treated with ATRA (4 μM) as well as AMPKα inhibitor (CC, 5 μM) for 24 h, proliferation rate of A7r5 was detected by MTS (*n* = 6). **(D)** Representative images of EdU staining. EdU (in red) stained the regions of cell proliferation; Hoechst33342 (in blue) stained the nuclei. **(E)** Percentage of EdU positive cells of A7r5 (*n* = 3). **(F)** HASMCs were, respectively, transfected with targeting AMPKα1/2 siRNA and negative control (NC) siRNA for 24 h, and then the expression levels of p-AMPKα, AMPKα, and p-MTOR were assessed by western blotting. **(G)** Relative expression levels of p-AMPKα and total-AMPKα (*n* = 3). **(H)** Relative expression levels of p-MTOR. **(I)** Proliferation rate of HASMC was detected by MTS (*n* = 6). **(J)** Representative images of EdU staining. **(K)** Percentage of EdU positive cells of HASMC (*n* = 3). **(L)** After respective transfection with AMPKα1/2 siRNA and NC siRNA, the migratory ability of HASMCs were detected with Transwell assays. **(M)** The number of cells in each field of view (*n* = 5). Data were shown as mean ± SD. ^∗^*p* < 0.05, ^∗∗^*p* < 0.01.

To further confirm whether activation of AMPKα is associated with the anti-proliferative and anti-migratory effects of ATRA on VSMC, AMPKα was inhibited by AMPKα inhibitor or siRNA. As shown in Figure 4A,B, treatment with ATRA (4 μM) alone significantly enhanced the phosphorylation level of AMPKα, while treatment with CC alone significantly reduced AMPKα phosphorylation without alteration of total expression of AMPKα. Co-treatment with CC and ATRA significantly reduced the phosphorylation level of AMPKα at Thr172 which was enhanced by ATRA. Consequently, as shown in Figure 4C–E, inhibition of AMPKα increased the cell proliferative ratio (% of Control group) of ATRA-treated A7r5 cells [ATRA (75.53 ± 4.36) *vs*. ATRA+CC (90.98 ± 5.16)], and significantly enhanced the EdU positive cell ratio of [ATRA (3.36 ± 0.369) *vs*. ATRA+CC (15.47 ± 0.729)]. Next, we inhibited AMPKα in HASMC by transfection with a target human AMPKα1 and AMPKα2 siRNA mix, and then detected cell proliferation by MTS and EdU staining and measured cell migration by Transwell assay. As shown in Figure 4F,G, treatment with ATRA alone significantly enhanced the phosphorylation level of AMPKα in the HASMC cells transfected with NC siRNA, however, the activation effects of ATRA was abolished in the VSMC transfected with targeting AMPKα1/2 siRNA. As shown in Figure 4F,H, the activation of mTOR was also rescued by AMPKα1/2 siRNA in ATRA treated-HASMCs. Consequently with AMPKα inhibitor, knockdown of AMPKα1/2 increased the cell proliferative ratio (Figure 4I) of ATRA-treated HASMC cells [HASMC^NC^+ATRA (44.6 ± 3.57) *vs*. HASMC^AMPKα-siRNA^+ ATRA (71.93 ± 2.74)], and significantly enhanced the EdU positive cell ratio (Figure 4J,K) of ATRA-treated HASMC cells [HASMC^NC^+ATRA (10.32 ± 1.069) *vs*. HASMC^AMPKα-siRNA^ +ATRA (24.99 ± 1.089)]. Then, we further investigated whether ATRA inhibited migration of HASMC through activation of AMPK. As shown in Figure 4L,M, we found that knockdown of AMPKα1/2 increased the cell migratory ratio of ATRA-treated HASMC cells [HASMC^NC^+ ATRA (158.52 ± 17.34) *vs*. HASMC^AMPKα-siRNA^+ ATRA (192.13 ± 12.28)]. The above findings indicated that the inhibitory effects of ATRA on VSMC proliferation and migration were due to, at least in part, the activation of AMPK.

### Molecular Simulations for the Interaction of ATRA With AMPK

**FIGURE 5:**
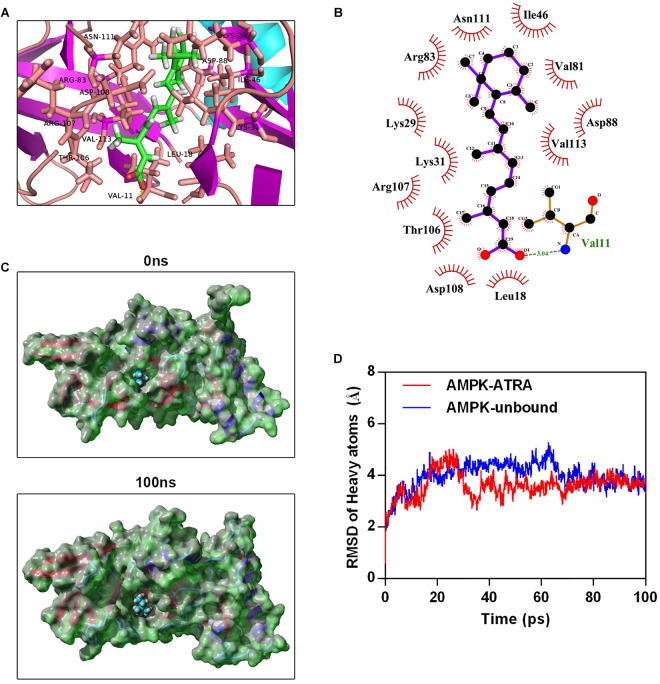
Molecular simulations for the interaction of ATRA with AMPK. **(A)** Three dimensional crystal structure of ATRA in complex with AMPK (PDB ID: 4QFR). ATRA are shown in green, and the hydrogen bonds are indicated by the yellow line. **(B)** Two dimensional crystal structure of ATRA in complex with AMPK. **(C)** Surface presentation of the AMPK-ATRA complex crystal structure at 0 ns and 100 ns. **(D)** Plots of root mean square deviation (RMSD) of heavy atoms of AMPK unbound (blue) and AMPKα-ATRA complex (red).

To explore the interaction between ATRA and AMPK, we performed molecular docking by using Autodock vina (Trott and Olson, 2010). The Molecular docking between AMPK agonist (A769662) and AMPK was also performed. The binding energy of AMPK-ATRA complex was -7.91 kcal/mol. The three dimensional and two-dimensional binding conformation of AMPK-ATRA complex are presented in Figure 5A,B, respectively. We found a hydrogen bond was formed between Val11 of AMPK and ATRA. The distance of hydrogen bond between AMPK and ATRA was 3.04 Å. It was also observed that ATRA interacted with Asn111, Ile46, Arg83, Lys29, Lys31, Thr106, Asp108, Leu18, Val81, Asp88, and Val113 *via* van der Waals force. The best conformation of AMPK-ATRA was taken as the start conformation for MD simulation via YASARA (Land and Humble, 2018). The surface visualization models of AMPK-ATRA complex are shown in Figure 5C. ATRA steadily presented at the center of AMPK binding site until the end of MD simulation. Figure 5D demonstrates the evolution of heave atoms RMSD of the complex concerning the minimized structure. The heave atoms RMSD track of AMPK-ATRA complex rose from 0.6 to 5 Å during the first 26 ns, declined from 5 to 2.7 Å during 26 to 44 ns, and then fluctuated around 3.5 Å during last 50 ns (Figure 5D, red line). The heavy atoms RMSD track of unbound AMPK raised from 0.6 to 5 Å during the first 20 ns, fluctuated between 4 and 5 Å during 20 to 65 ns, then fluctuated around 3.5 Å during last 35 ns (Figure 5D, blue line). These results suggest a strong binding between the kinase domain of AMPK and ATRA, indicating that ATRA could directly target AMPKα.

## Discussion

ATRA is a natural derivative of vitamin A, and has a number of beneficial health actions including anti-atherosclerosis and anti-neointimal hyperplasia. In this study, we demonstrated the anti-neointimal hyperplasia effect of ATRA in common carotid artery ligation mouse model, an animal model designed to investigate the anti-neointimal hyperplasia actions of the drug. Our data showed that ATRA inhibited neointimal hyperplasia in a dose-dependent manner, which is in agreement with the results of previous studies that showed that ATRA inhibited neointimal hyperplasia after balloon angioplasty in atherosclerotic rabbit (Wiegman et al., 2000). Proliferation and migration of VSMC is a central pathological mechanism of neointimal hyperplasia. In this study, we demonstrated the anti-neointimal hyperplasia effect and working mechanism of ATRA in A7r5 and HASMC—two cell models for investigating the anti-atherosclerosis actions of drug (Han et al., 2017; Zhou et al., 2018). Our data showed that ATRA inhibited A7r5 and HASMC VMSCs proliferation and migration in a dose-dependent manner which is in agreement with the results of previous studies (Tran-Lundmark et al., 2015). Endothelial cells are the barrier of blood vessel; endothelial dysfunction plays a key role in the formation of atherosclerosis (Dong et al., 2017). Thus, we speculated that if ATRA reduced the cell viability of endothelial cells when in inhibited VSMC proliferation. Interestingly, a previous study has found that ATRA (1 μM) reduced the proliferation of smooth muscle cell, while enhancing endothelial cell proliferation (Bilbija et al., 2014). It was also found that ATRA increased the proliferation of endothelial cell and induced angiogenesis (Saito et al., 2007). Therefore, we speculate that ATRA might reduce the proliferation without inducing the endothelial cell injury. Cell injury plays a crucial role in atherosclerosis (Saito et al., 2007; Gimbrone and Garcia-Cardena, 2016). Therefore, we further investigated the cell death or apoptosis in VSMC to determine whether ATRA inhibited cell proliferation of VSMC by inducing cell injury. We found that ATRA could not induce cell death or apoptosis. Our data suggest that ATRA suppressed cell proliferation without inducting cell injury.

AMPK is widely involved in the development as well as progression of atherosclerosis (Almabrouk et al., 2014). AMPK activation by ATRA has been reported in ovarian cancer, skeletal muscle cells and endothelial cells (Lee et al., 2008; Ishijima et al., 2015; Kim et al., 2015), while the effect of ATRA on AMPK in VSMCs is unclear. In present study, we found that ATRA significantly increased the phosphorylation of AMPKα of the common carotid artery at Thr172 in a dose-dependent manner. Interestingly, we found p-AMPK-positive staining was mostly observed in media and adventitia rather than in neointima. This result was in agreement with the finding of a precious study (Cacicedo et al., 2011). We speculated that low activation level of AMPK should be an important characteristic of migrated and over-proliferative VSMCs. We also found ATRA significantly increased the phosphorylation of AMPKα at Thr172 in a dose- and time-dependent manner. These data suggested that AMPK might be the pharmacological target of ATRA and activation of AMPK by ATRA may be a novel treatment strategy for atherosclerosis. There is no AMPK activator drug for clinical use so far. The most important advantage of ATRA in comparison with other small molecule agonists of AMPK is that ATRA is an FDA-approved drug (Bhat-Nakshatri et al., 2013). In addition, the essential involvement of AMPK has been detected in a series of pathophysiology processes, including glucose metabolism, fatty acid metabolism, inflammation and autophagy (Steinberg and Schertzer, 2014; Day et al., 2017). Our findings provide a rationale for using ATRA in regulating these pathophysiology processes. In addition, some known mechanism of ATRA on VSMC proliferation could be explained by AMPK activation. For example, it was reported that ATRA inhibited the proliferation of VSMC though overexpression of Klf4 (Wang et al., 2008), while activation of AMPK could enhance the expression level of Klf4 (Sunaga et al., 2016).

It has been demonstrated that activation of AMPK inhibited the activity of mTOR signaling in VSMC (Ke et al., 2016). Thus, we further determined whether ATRA inhibited the activation of mTOR signaling in VSMC. We found that treatment with ATRA dose-dependently reduced the phosphorylation levels of mTOR and its downstream molecules, p70S6K and 4EBP1. These results suggest that ATRA should activate AMPK and inhibit the activity of mTOR signaling. Our findings were in agreement with the results of previous studies that ATRA inhibited mTOR activation in myeloid leukemia cells and liver fibrosis (Sharvit et al., 2013; Chen et al., 2014), while another study demonstrated that ATRA enhanced mTOR activity in adipose-derived stromal cells (Glanz et al., 2016). Differential roles of ATRA in mTOR signaling may be due to cell type specificity.

Next, we determined the involvement of AMPK in the anti-proliferative and anti-migratory action of ATRA by inhibition of AMPK using CC, an inhibitor of AMPKα (Liu et al., 2014). We found that the anti-proliferative effects of ATRA were partly counteracted by CC. Inhibition of AMPK rescued the proliferation of VSMC that was inhibited by ATRA. CC is a non-specific inhibitor of AMPK and has many off-target effects. Thus, we further determined the role of AMPK in the pharmacological effects of ATRA by siRNA targeting AMPKα1/2. AMPKα1 and AMPKα2 are two subunits of the AMPKα, and both AMPKα1 and α2 are expressed in VSMC (Rubin et al., 2005). Thus, we knocked down the two subunits by siRNA and found that the proliferation and migration of VSMC were partly rescued by AMPK inhibition. These results indicate that AMPK involves in the anti-proliferative and anti-migratory action of ATRA. Meanwhile, we found inhibition of AMPK just partly abolished the inhibitory effects of ATRA on the proliferation and migration of VSMC, indicating that ATRA should exerts these pharmacologic actions through AMPK and other potential targets, such as retinoic acid receptors, a known ATRA target.

Next, we further investigated whether ATRA target AMPK directly, and performed molecular docking and MD simulation to determine the mechanism of interaction of ATRA and AMPK. The binding energy between ATRA and AMPK was -7.91 kcal/mol, indicating good binding ability. A hydrogen bond was formed between Val11 of AMPK and ATRA. Several direct AMPK activators have hydrogen bond that interacts with Val11 (Xiao et al., 2013; Huang et al., 2017). In addition, ATRA interacted with Asn111, Ile46, Arg83, Lys29, Lys31, Thr106, Asp108, Leu18, Val81, Asp88 and Val113 of AMPK *via* van der Waals force. These residues are the key residues in the agonist binding domain of AMPK, which interact with AMPK agonists including PF-06409577 and A-769662 (Cool et al., 2006; Cameron et al., 2016). Moreover, MD simulation of the AMPK-ATRA complex indicated that binding conformations of ATRA with AMPK is stable. In this study, we predicted the binding ability of ATRA and AMPK in the binding site of the AMPK activator, A-769662. Thus, we speculated that ATRA should activate AMPK through a mechanism similar to that of A-769662. It was reported that A-769662 enhanced the phosphorylation of Thr172 through inhibiting dephosphorylation of Thr172 (Goransson et al., 2007). The binding site of A-769662 is between AMPK α and β. When A-769662 binds with AMPK α and β heterodimer, it would increase the interaction between AMPK α and β, stabilize this complex, and prevent Thr172 against protein phosphatases (Xiao et al., 2013). These results indicated that ATRA targets AMPKα directly. ATRA is a potential AMPK agonist. The major limitation of this study is that owing to the lack of the appropriate experimental conditions we only investigate the interaction of AMPK and ATRA by computer simulation without confirming this by experiment. We intend to address this in future research.

In summary, we report that ATRA could suppress neointimal hyperplasia and inhibit proliferation and migration of VSMCs. These effects were partially due to the activation of AMPK. Our findings provide novel insights into the anti-neointimal hyperplasia mechanisms of ATRA and implicate the therapeutic potential of ATRA in neointimal hyperplasia management.

## Ethics Statement

This study was carried out in accordance with the recommendations of the guidelines of the Institutional Animal Care and Use Committee of The Second Affiliated Hospital of Guangzhou Medical University. The protocol was approved by the Institutional Animal Care and Use Committee of The Second Affiliated Hospital of Guangzhou Medical University.

## Author Contributions

JZ supervised the entire work and performed the cell cultures and proliferation assays. BD and XJ performed the cell cultures and animal experiments. WJ performed histopathology assay. MC and YT performed the western blot assay. NL and SZ performed the molecular docking and simulation of molecular dynamics. BL and GH analyzed the data. BL and SL conceived and designed the experiments and critically revised the manuscript. All authors discussed the results and contributed to manuscript writing.

## Conflict of Interest Statement

The authors declare that the research was conducted in the absence of any commercial or financial relationships that could be construed as a potential conflict of interest.

## Supplementary Material

FIGURE S1 ATRA inhibited the proliferation and migration of HASMC. **(A)** HASMC cells were incubated with indicated doses of ATRA (1, 2 as well as 4 μM) for 24 h, followed by the MTS assay to determine the proliferation of HASMC (*n* = 6). **(B)** Representative images of EdU staining. EdU (in red) stained the regions of cell proliferation; Hoechst33342 (in blue) stained the nuclei. **(C)** Percentage of EdU positive cells of HAMSC (*n* = 3). **(D)** The expressions of CyclinD1 and CyclinD3 were tested via western blotting. **(E)** Relative levels of CyclinD1 and CyclinD3 (*n* = 3). **(F)** HASMC cells were treated with different concentrations of ATRA (1, 2, and 4 μM), and tested by performing Transwell assays for 12 h. **(G)** The number of cells in each field of view (*n* = 5). Data were presented as Mean ± SD. ^∗^*p* < 0.05, ^∗∗^*p* < 0.01 compared with the Control group.

FIGURE S2 ATRA inhibited the PDGF-BB-induced VSMC proliferation and migration. **(A,B)** A7r5 **(A)** and HASMC **(B)** cells were treated by PDGF-BB (20 ng/mL) with or without ATRA (1, 2 as well as 4 μM) for 24 h, followed by the MTS assay to determine the proliferation of VSMC (*n* = 6). **(C)** A7r5 and HASMC cells were treated with PDGF-BB (20 ng/mL) with or without ATRA (1, 2 as well as 4 μM), and subjected by performing Transwell assays for 12 h. **(D,E)** The number of A7r5 **(D)** and HASMC **(E)** in each field of view (*n* = 5). Data are presented as mean ± SD. ^∗^*p* < 0.05, ^∗∗^*p* < 0.01 compared with the PDGF-BB group.

FIGURE S3 ATRA did not induced cell injury of VSMC. **(A)** Cell dead were measured by LIVE/DEAD staining. Live cells were labeled with green, dead cells were marked with red. **(B)** The expressions of Bax, Bcl-xl, Pro-caspase-3, and Cleaved-caspase-3 were tested by western blotting. **(C)** Relative levels of Bax, Bcl-xl, Pro-caspase-3, and Cleaved-caspase-3 expression (*n* = 3). Data were presented as mean ± SD.

FIGURE S4 ATRA and AICAR inhibited the proliferation and migration of HASMC. **(A)** HASMC cells were, respectively, incubated with ATRA (4 μM) and AICAR (1 mM) for 6 h, followed by western blotting to determine the expression levels of p-AMPKα and AMPKα (*n* = 3). **(B)** Relative expression level of p-AMPK (*n* = 3). **(C)** HASMC cells were, respectively, treated with ATRA (4 μM) and AICAR (1 mM) for 24 h, then the proliferation of HASMC cells were measured by MTS (*n* = 6). **(D)** HASMC cells were treated with ATRA (4 μM) and AICAR (1 mM), and tested by performing Transwell assays for 12 h. **(E)** The number of cells in each field of view (*n* = 5). Data are presented as mean ± SD. ^∗^*p* < 0.05, ^∗∗^*p* < 0.01 compared with the Control group.

## Abbreviations

4EBP1: 4E-binding protein 1
AMPK: AMP-activated protein kinase
ATRA: all-*trans*-retinoic acid
CC: compound C
Klf4: Kruppel-like factor 4
MD: molecular dynamics
mTOR: mammalian target of rapamycin
p70S6K: p70 S6 kinase
RMSD: root-mean-square deviation
VSMC: vascular smooth muscle cell
