# Supplementary figures and images for: All-Trans-Retinoic Acid Suppresses Neointimal Hyperplasia and Inhibits Vascular Smooth Muscle Cell Proliferation and Migration via Activation of AMPK Signaling Pathway
Source: Front Pharmacol. 2019 May 9;10:485. doi: 10.3389/fphar.2019.00485 (PMC6521230; doi:10.3389/fphar.2019.00485)

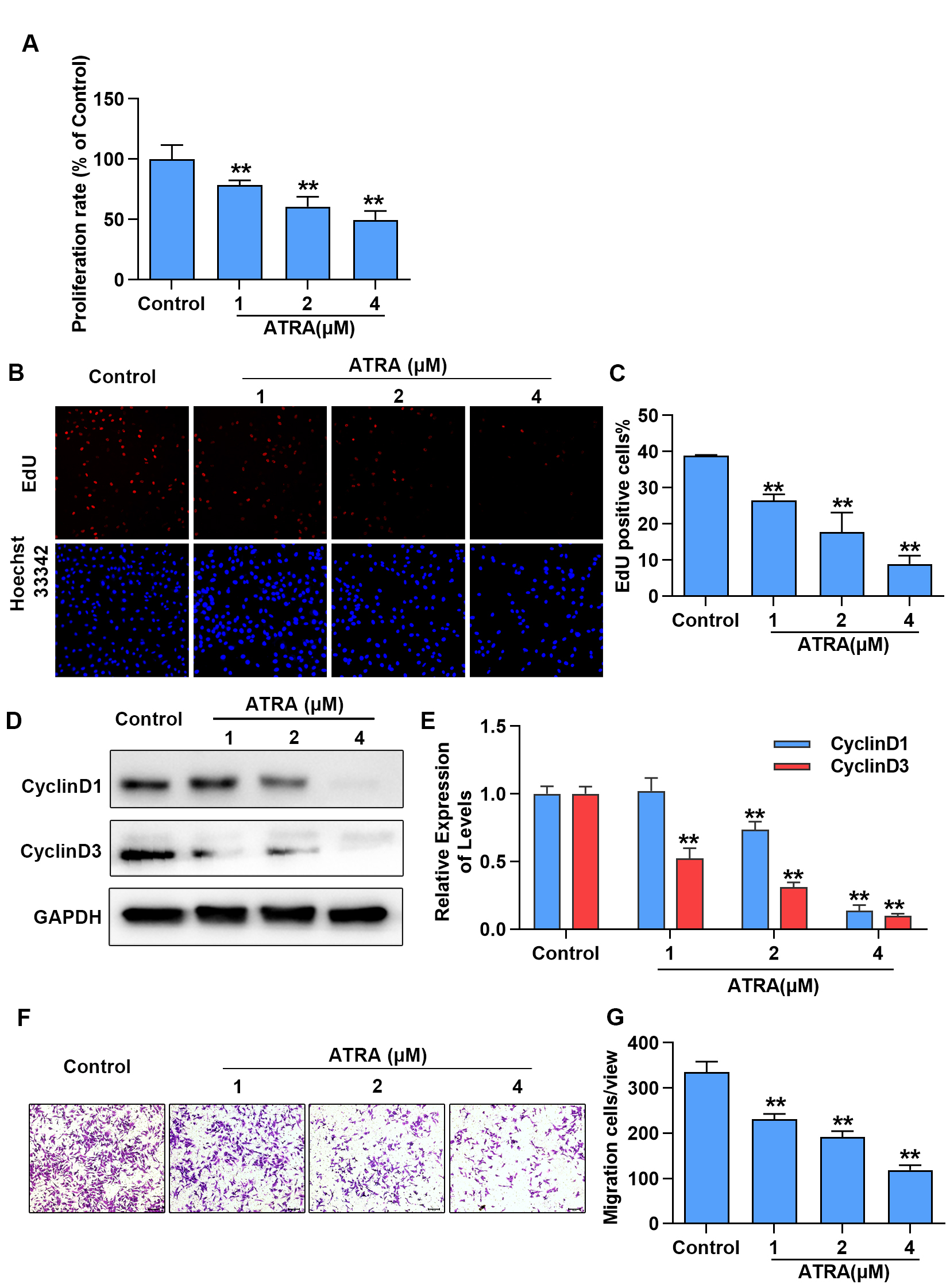

Supplement: FIGURE S1 — ATRA inhibited the proliferation and migration of HASMC. (A) HASMC cells were incubated with indicated doses of ATRA (1, 2 as well as 4 μM) for 24 h, followed by the MTS assay to determine the proliferation of HASMC (n = 6). (B) Representative images of EdU staining. EdU (in red) stained the regions of cell proliferation; Hoechst33342 (in blue) stained the nuclei. (C) Percentage of EdU positive cells of HAMSC (n = 3). (D) The expressions of CyclinD1 and CyclinD3 were tested via western blotting. (E) Relative levels of CyclinD1 and CyclinD3 (n = 3). (F) HASMC cells were treated with different concentrations of ATRA (1, 2, and 4 μM), and tested by performing Transwell assays for 12 h. (G) The number of cells in each field of view (n = 5). Data were presented as Mean ± SD. ∗p < 0.05, ∗∗p < 0.01 compared with the Control group. [file Data_Sheet_1.zip › Supplementary material/Figure S1_V1.JPEG]

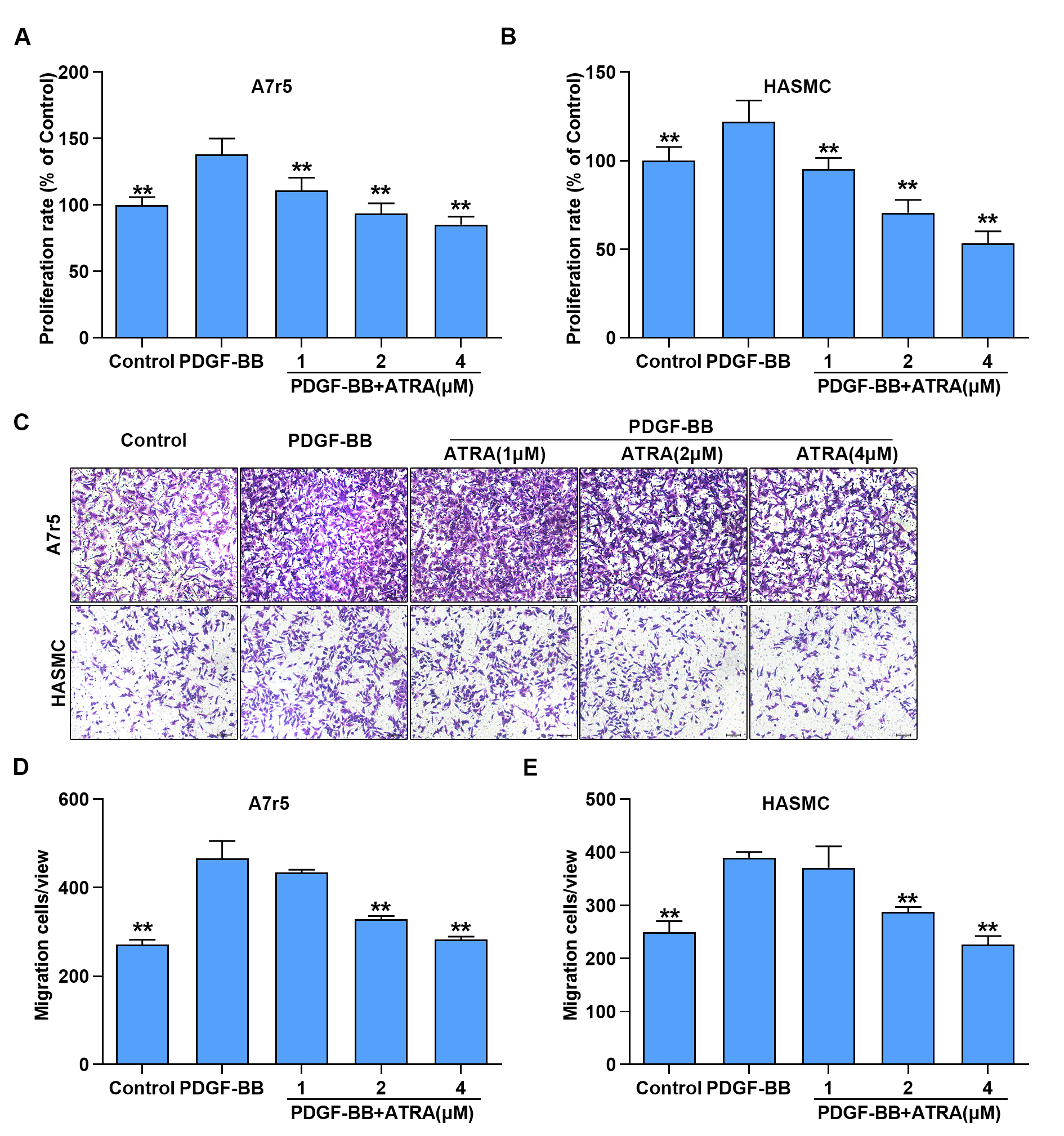

Supplement: FIGURE S1 — ATRA inhibited the proliferation and migration of HASMC. (A) HASMC cells were incubated with indicated doses of ATRA (1, 2 as well as 4 μM) for 24 h, followed by the MTS assay to determine the proliferation of HASMC (n = 6). (B) Representative images of EdU staining. EdU (in red) stained the regions of cell proliferation; Hoechst33342 (in blue) stained the nuclei. (C) Percentage of EdU positive cells of HAMSC (n = 3). (D) The expressions of CyclinD1 and CyclinD3 were tested via western blotting. (E) Relative levels of CyclinD1 and CyclinD3 (n = 3). (F) HASMC cells were treated with different concentrations of ATRA (1, 2, and 4 μM), and tested by performing Transwell assays for 12 h. (G) The number of cells in each field of view (n = 5). Data were presented as Mean ± SD. ∗p < 0.05, ∗∗p < 0.01 compared with the Control group. [file Data_Sheet_1.zip › Supplementary material/Figure S2_V1.JPEG]

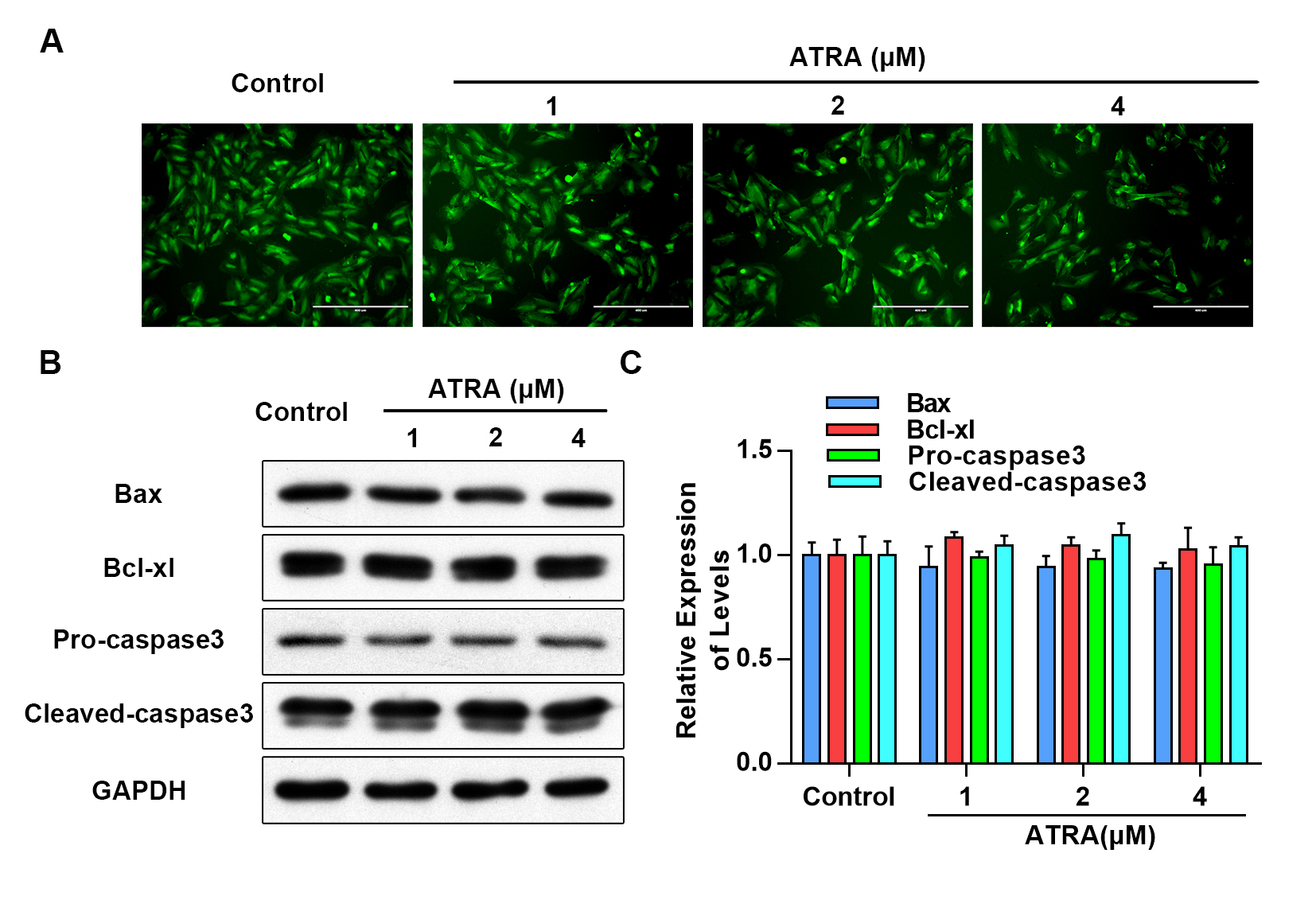

Supplement: FIGURE S1 — ATRA inhibited the proliferation and migration of HASMC. (A) HASMC cells were incubated with indicated doses of ATRA (1, 2 as well as 4 μM) for 24 h, followed by the MTS assay to determine the proliferation of HASMC (n = 6). (B) Representative images of EdU staining. EdU (in red) stained the regions of cell proliferation; Hoechst33342 (in blue) stained the nuclei. (C) Percentage of EdU positive cells of HAMSC (n = 3). (D) The expressions of CyclinD1 and CyclinD3 were tested via western blotting. (E) Relative levels of CyclinD1 and CyclinD3 (n = 3). (F) HASMC cells were treated with different concentrations of ATRA (1, 2, and 4 μM), and tested by performing Transwell assays for 12 h. (G) The number of cells in each field of view (n = 5). Data were presented as Mean ± SD. ∗p < 0.05, ∗∗p < 0.01 compared with the Control group. [file Data_Sheet_1.zip › Supplementary material/Figure S3_V1.JPEG]

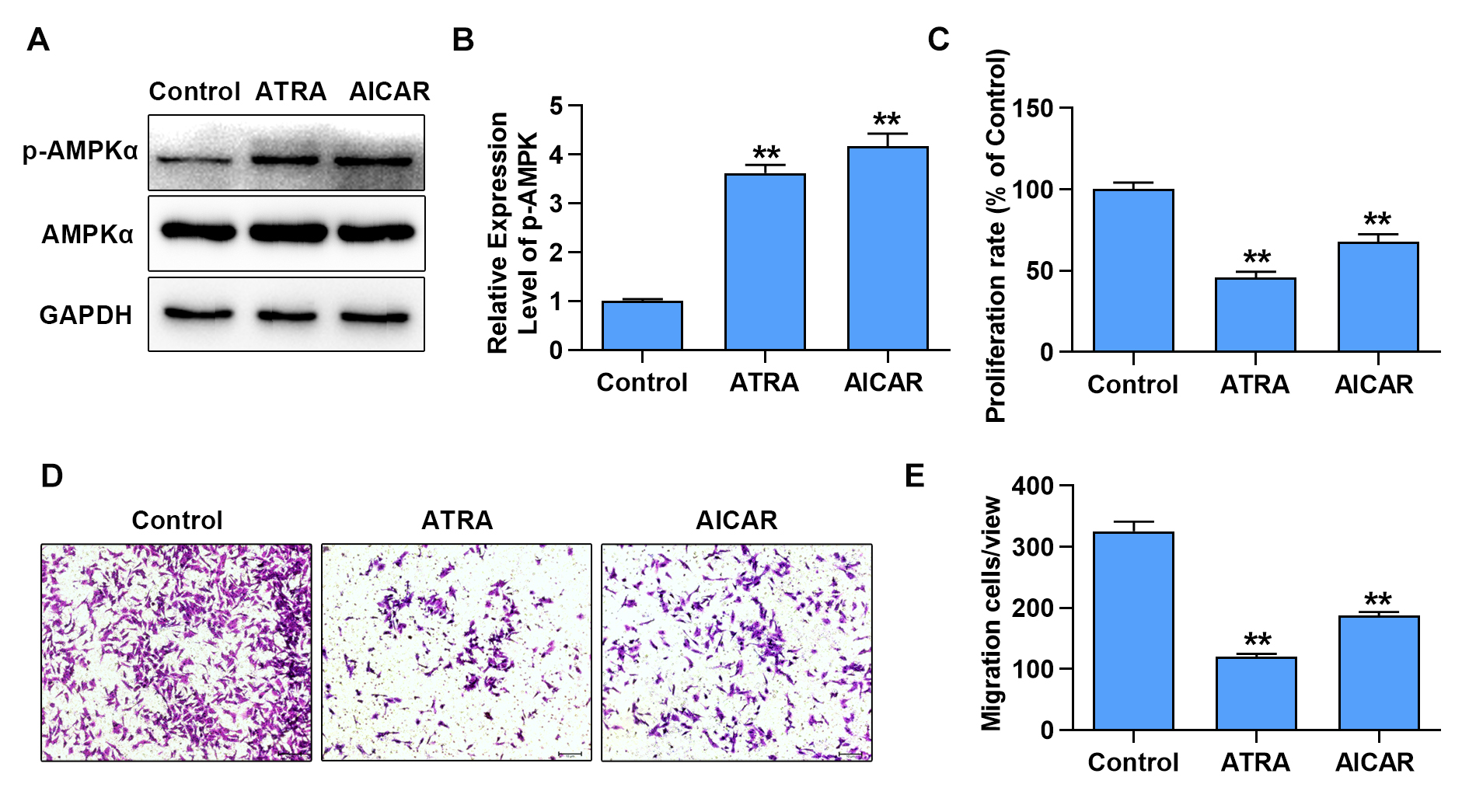

Supplement: FIGURE S1 — ATRA inhibited the proliferation and migration of HASMC. (A) HASMC cells were incubated with indicated doses of ATRA (1, 2 as well as 4 μM) for 24 h, followed by the MTS assay to determine the proliferation of HASMC (n = 6). (B) Representative images of EdU staining. EdU (in red) stained the regions of cell proliferation; Hoechst33342 (in blue) stained the nuclei. (C) Percentage of EdU positive cells of HAMSC (n = 3). (D) The expressions of CyclinD1 and CyclinD3 were tested via western blotting. (E) Relative levels of CyclinD1 and CyclinD3 (n = 3). (F) HASMC cells were treated with different concentrations of ATRA (1, 2, and 4 μM), and tested by performing Transwell assays for 12 h. (G) The number of cells in each field of view (n = 5). Data were presented as Mean ± SD. ∗p < 0.05, ∗∗p < 0.01 compared with the Control group. [file Data_Sheet_1.zip › Supplementary material/Figure S4_V1.JPEG]
